# Frailty and Sarcopenia as Predictors of Functional Recovery After Total Hip and Knee Arthroplasty: A Narrative Review

**DOI:** 10.3390/jcm15103578

**Published:** 2026-05-07

**Authors:** Man Hung, Annabella Jensen, Isabella Strickler, Sharon Vu, Eric S. Hon, Avianna Arapovic, Mouhanad M. El-Othmani

**Affiliations:** 1 College of Dental Medicine, Roseman University of Health Sciences, South Jordan, UT 84095, USA; 2 Department of Orthopaedic Surgery Operations, University of Utah, Salt Lake City, UT 84108, USA; 3 William Beaumont School of Medicine, Oakland University, Rochester, MI 48309, USA; 4 Lake Erie College of Osteopathic Medicine, Erie, PA 16509, USA; 5 Department of Economics, University of Chicago, Chicago, IL 60637, USA; 6 Idaho College of Osteopathic Medicine, Meridian, ID 83642, USA; 7 Department of Orthopaedic Surgery, Corewell Health, Royal Oak, MI 48073, USA; 8 Warren Alpert Medical School, Brown University, Providence, RI 02903, USA

**Keywords:** frailty, sarcopenia, total joint arthroplasty, total knee arthroplasty, physical therapy utilization, functional recovery

## Abstract

**Introduction:** Total hip arthroplasty (THA) and total knee arthroplasty (TKA) volumes continue to rise, yet postoperative functional recovery and physical therapy (PT) utilization remain highly variable. Frailty and sarcopenia capture biological vulnerability beyond chronological age and may better explain heterogeneity in rehabilitation needs and recovery trajectories. **Methods:** We conducted a qualitative narrative review guided by SANRA recommendations. PubMed, Scopus, Web of Science, CINAHL, and Google Scholar were searched for English-language studies (January 2000–December 2025) involving adults undergoing THA/TKA that assessed preoperative frailty and/or sarcopenia and reported postoperative functional or rehabilitation-related outcomes. Data were extracted and synthesized thematically without quantitative pooling. **Results:** Thirty-four studies met inclusion criteria. Despite heterogeneous definitions and measures, both frailty and sarcopenia were consistently associated with slower early mobility, poorer functional recovery, longer hospital stays, and higher rates of non-home discharge. Frailty showed stronger links to post-acute care use and healthcare costs, whereas sarcopenia was more closely tied to impaired early function, delayed ambulation, and greater reliance on assistive devices. Frailty may improve after arthroplasty in some patients, while sarcopenia often represents a more persistent, muscle-specific limitation. Direct reporting of PT dose was uncommon, with only a minority of included studies explicitly reporting physical therapy frequency, duration, or intensity, limiting the ability to assess dose–response relationships. **Conclusions:** Preoperative frailty and sarcopenia are clinically meaningful predictors of functional recovery and rehabilitation utilization after THA/TKA and support vulnerability-informed discharge planning and stratified rehabilitation pathways. Future prospective studies should standardize vulnerability assessment and directly quantify PT dose, setting, and patient-centered outcomes to inform and test vulnerability-guided rehabilitation strategies, including prehabilitation.

## 1. Introduction

Total hip arthroplasty (THA) and total knee arthroplasty (TKA) are among the most commonly performed elective surgical procedures worldwide, with utilization expected to increase due to population aging and rising prevalence of osteoarthritis. Although advances in surgical technique and perioperative care have improved short-term outcomes, postoperative functional recovery remains highly variable [1]. Physical therapy (PT) is central to restoring mobility and function and facilitating safe discharge after THA and TKA, yet PT utilization, intensity, and duration vary widely across patients.

Rehabilitation planning after arthroplasty is often guided by chronological age, procedure type, and institutional care pathways [2]. However, these factors only partially explain why some patients recover rapidly with minimal therapy while others require prolonged or higher-intensity services. Biological vulnerability—the capacity to withstand perioperative stress and recover—may be a more informative determinant of postoperative trajectories and rehabilitation demand [3].

Frailty and sarcopenia are complementary constructs that operationalize biological vulnerability. Frailty is a multidimensional syndrome characterized by reduced physiologic reserve and resilience to stressors. In THA and TKA populations, preoperative frailty has been associated with longer hospital stays, higher complication rates, non-home discharge, and delayed functional recovery [4]. Sarcopenia, defined by low skeletal muscle mass and strength, is prevalent in older adults and increasingly recognized in arthroplasty cohorts [5]. Measures of sarcopenia and muscle quality have been linked to impaired mobility, smaller strength gains, and poorer functional outcomes after joint replacement [6].

Despite growing recognition of frailty and sarcopenia as predictors of surgical outcomes, their implications for postoperative PT utilization are less clearly synthesized. Rehabilitation demand is influenced not only by surgical factors but also by patients’ capacity to participate in, tolerate, and respond to therapy [7]. Frailty and sarcopenia may therefore shape discharge destination, post-acute care use, and the intensity and duration of PT. However, the existing literature is fragmented across disciplines, employs heterogeneous definitions and measurement tools, and often reports rehabilitation-related outcomes indirectly [8].

Given these limitations, a narrative review is well suited to integrate diverse evidence streams, critically appraise conceptual and methodological variability, and explore the implications of frailty and sarcopenia for rehabilitation planning following THA and TKA.

Therefore, the objectives of this review were to:

(1) Synthesize evidence on preoperative frailty and sarcopenia as predictors of postoperative functional recovery after THA and TKA;
(2) Examine their relationship with physical therapy utilization and post-acute care needs;
(3) Discuss implications for rehabilitation planning and future research.

## 2. Methods

This narrative review was conducted in accordance with the Scale for the Assessment of Narrative Review Articles (SANRA) recommendations [9].

### 2.1. Review Design

A qualitative narrative review methodology was selected to synthesize heterogeneous evidence across orthopedics, geriatrics, rehabilitation, and health services research. Given variability in definitions, outcome measures, and study designs, a thematic synthesis approach was used rather than quantitative aggregation or meta-analysis.

### 2.2. Literature Search Strategy

A comprehensive literature search was performed to identify studies examining frailty and/or sarcopenia in patients undergoing THA and TKA and reporting postoperative functional recovery and/or rehabilitation-related outcomes. Electronic databases included PubMed, Scopus, Web of Science, CINAHL, and Google Scholar. Articles published from January 2000 through December 2025 were considered. The timeframe was selected to reflect contemporary arthroplasty techniques and rehabilitation practices while capturing foundational studies in this field. Search strategies combined keywords and Boolean operators, including terms such as: (“frailty” OR “frail”) OR (“sarcopenia”) AND (“total hip arthroplasty” OR “total knee arthroplasty”) AND (“rehabilitation” OR “physical therapy” OR “functional recovery”). Reference lists of key articles were also screened to identify additional relevant studies.

### 2.3. Eligibility Criteria

Studies were eligible if they included adults undergoing THA and/or TKA, assessed frailty and/or sarcopenia preoperatively, and reported postoperative functional recovery and/or rehabilitation-related outcomes. Studies were excluded if they involved non-arthroplasty populations, were non-peer-reviewed, lacked relevant functional or rehabilitation outcomes, or were not published in English.

Titles and abstracts were screened for relevance, followed by full-text review. Reasons for exclusion included absence of relevant outcomes, inappropriate population, or insufficient methodological detail.

### 2.4. Data Extraction and Narrative Synthesis

Extracted data included study design and population characteristics; definitions and measurement tools; timing of assessment; functional recovery outcomes; and rehabilitation-related outcomes, including PT utilization and discharge disposition. Evidence was synthesized thematically to identify consistent patterns, areas of disagreement, and clinically meaningful implications for PT practice. No quantitative pooling of data was performed. No quantitative pooling or meta-analysis was performed.

### 2.5. Critical Appraisal Approach

**Figure 1:**
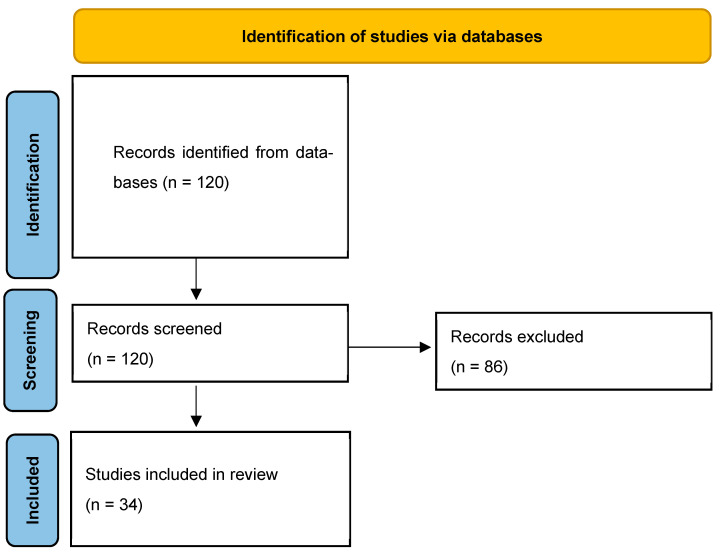
Study Selection Flow Diagram.

Formal risk-of-bias assessment tools were not applied, consistent with narrative review methodology. Instead, methodological strengths and limitations of included studies were discussed qualitatively, focusing on heterogeneity of definitions, variability in rehabilitation outcome reporting, and confounding. Limitations were highlighted to inform interpretation and future research. The study selection process is summarized in Figure 1.

## 3. Frailty and Sarcopenia in THA and TKA

### 3.1. Definitions and Operationalization

Although frailty and sarcopenia are often discussed together in the arthroplasty literature, they represent distinct clinical conditions that are operationalized using different criteria across studies. Frailty reflects increased physiological vulnerability due to aging-associated decline in multiple organ systems, compromising an individual’s ability to respond to stressors [10]. In contrast, sarcopenia represents a more specific syndrome characterized by loss of skeletal muscle mass and function. For rehabilitation planning, frailty broadly reflects reserve and resilience, whereas sarcopenia highlights muscle-specific capacity relevant to mobility and strengthening. Importantly, frailty and sarcopenia frequently overlap in clinical populations, as muscle weakness contributes to frailty phenotypes; however, they remain distinct constructs. This overlap may contribute to variability in reported associations depending on how each condition is defined and measured across studies.

### 3.2. Measurement Tools Used in Arthroplasty Populations

In arthroplasty populations, frailty is most commonly assessed using tools such as the Clinical Frailty Scale (CFS), the Fried frailty phenotype, or the modified frailty index (mFI). There is currently no consensus on which tool is optimal for routine preoperative assessment. The literature indicates that the mFI is the most frequently used frailty measure in arthroplasty research [11], whereas feasibility-oriented work suggests that the CFS may be among the most practical options for routine preoperative use [12]. These assessments vary in scope and methodology: the CFS ranks patients on a 1–9 scale based on overall fitness and dependence in daily activities (9 indicating severe frailty or terminal illness) [13]; the Fried phenotype identifies frailty according to five physical criteria (unintentional weight loss, weakness, exhaustion, slowed gait, and low physical activity) [14]; and the mFI summarizes comorbidities and functional deficits to estimate perioperative risk [15], with higher scores indicating increasing frailty [16].

In contrast, sarcopenia assessment in THA and TKA patients typically begins with practical physical performance measures, such as grip strength, gait speed, and chair-rise tests, that are feasible in the preoperative setting [17]. International guidelines now emphasize muscle function as the primary criterion for case finding and diagnosis, as functional measures are more strongly associated with clinical outcomes than muscle mass alone [18,19,20]. Imaging may quantify muscle mass but is less used preoperatively because of cost and access limitations [17,21]. Consequently, many studies rely on functional or surrogate markers rather than comprehensive diagnostic workups [20]. This pattern reflects real-world barriers to formal sarcopenia diagnosis, including limited access to specialized equipment and inconsistent use of standardized criteria in routine practice [17]. Commonly used frailty and sarcopenia measures in THA and TKA populations, along with the domains captured, feasibility considerations, and rehabilitation relevance, are summarized in Table 1. Where available, clinically meaningful thresholds have been proposed to aid interpretation. For example, gait speed <0.8 m/s and reduced grip strength (e.g., <27 kg in men and <16 kg in women) are commonly used indicators of sarcopenia-related functional impairment. Similarly, higher CFS scores (≥5) generally reflect increasing frailty severity and vulnerability. While thresholds vary across studies, these values provide practical reference points for clinical application.

### 3.3. Clinical Relevance to Rehabilitation Planning

Understanding these differences in definition and operational assessment is important for rehabilitation planning because each measure captures distinct dimensions of risk and rehabilitation capacity. Frailty instruments reflect multisystem vulnerability (e.g., physiologic reserve, comorbidity burden, and dependence), which can inform anticipated therapy tolerance, discharge needs, and the likelihood of requiring post-acute services. In contrast, sarcopenia-focused assessments emphasize muscle strength and physical performance, highlighting specific impairments (e.g., weakness, slowed gait, difficulty rising from a chair) that are directly targetable through strengthening, balance training, and nutritional optimization. Together, these tools support more individualized rehabilitation planning by distinguishing patients who primarily need broader support and pacing from those who may benefit most from targeted neuromuscular and functional interventions.

## 4. Preoperative Frailty and Sarcopenia as Predictors of Postoperative Functional Recovery

### 4.1. Mobility Outcomes

Preoperative frailty and sarcopenia have emerged as important predictors of postoperative functional recovery following total joint arthroplasty [16,22,23]. Patients with sarcopenia undergoing primary TKA experience longer hospital stays and higher rates of postoperative complications [22,24], which may delay early mobilization and limit initial functional gains. In addition, postoperative quadriceps function is significantly impaired following TKA, with reductions in both muscle strength and voluntary activation [25], which can hinder early ambulation and gait recovery. Postoperative gait speed is reduced in patients with sarcopenia, further highlighting the impact of impaired muscle function on walking ability [26]. Similarly, frailty, regardless of the measurement tool used, predicts prolonged hospitalization and slower early recovery [3,16].

### 4.2. ADLs and Performance-Based Measures

Beyond mobility, both frailty and sarcopenia influence independence in activities of daily living (ADL) and other performance-based measures. An analysis that included complete frailty measures found that nearly 30% of patients undergoing orthopedic procedures had impaired instrumental ADL [16]. Frailty has also been linked to elevated short-term mortality after arthroplasty, reflecting reduced physiologic reserve [27]. However, frailty is increasingly recognized as a dynamic and potentially modifiable state rather than a fixed preoperative risk factor: one study found that patients classified as frail or vulnerable preoperatively showed improvements in frailty status after surgery, accompanied by significant gains in ADL such as walking, stair climbing, and independence with self-care [28]. Similarly, another prospective study of THA and TKA patients found that pre-frail patients often regained robustness, and some patients initially classified as frail were no longer considered frail after surgery, suggesting that joint replacement can improve frailty status in people with hip or knee osteoarthritis [29].

In contrast, sarcopenia represents a muscle-specific impairment that may continue to constrain functional independence after arthroplasty [30,31]. Patients with sarcopenia undergoing THA are at increased risk for falls and fragility fractures in the first one to two years after surgery, which can further impair ADL independence [24]. This contrast—potentially improving frailty versus persistent muscle impairment—helps explain why some patients’ global risk profile improves after surgery while their functional ceiling remains limited by weakness.

### 4.3. Recovery Trajectories and Delays

The impact of preoperative frailty and sarcopenia extends beyond immediate postoperative outcomes by influencing the trajectory of functional recovery. Higher frailty scores, including the mFI, have been associated with increased risk of severe postoperative complications and greater likelihood of discharge to post-acute care facilities [16,32], reflecting delayed or more complex recovery.

Emerging evidence suggests that incorporating simple physical performance measures, such as handgrip strength and gait speed, may improve identification of patients at risk for delayed functional recovery following arthroplasty [31]. These measures operationalize key functional components of sarcopenia [18,19,20], capturing impairments in muscle function that are closely linked to the pace of postoperative recovery. Consistent with this, sarcopenia was highly prevalent among patients undergoing THA in rehabilitation settings, where impairments in balance and gait independence are closely associated with sarcopenia severity [33]. Importantly, severe sarcopenia in patients undergoing THA has been associated with poorer hip function and worse patient-reported outcomes at six months postoperatively, indicating a slower and less complete recovery trajectory compared with non-sarcopenic patients [31]. Interventions targeting sarcopenia in the preoperative period, including prehabilitation and nutritional optimization, have been proposed as strategies to enhance functional gains and reduce delays [34].

Taken together, these findings underscore that preoperative identification and optimization of frailty and sarcopenia can shape the timing, pace, and completeness of postoperative functional recovery.

## 5. Implications for PT Utilization After THA and TKA

Frailty and sarcopenia appear to shape physical therapy utilization after THA and TKA, particularly discharge destination, post-acute service use, and associated costs. Across studies, frail or sarcopenic patients are more likely to require non-home discharge (e.g., inpatient rehabilitation or skilled nursing facilities) than non-frail counterparts. In a large single-center cohort of THA and TKA patients, higher CFS scores were associated with longer hospital length of stay and higher rates of inpatient rehabilitation admission [35]. Key studies examining associations between frailty or sarcopenia and postoperative functional recovery, discharge disposition, post-acute care utilization, and other rehabilitation-relevant outcomes are summarized in Table 2. Across heterogeneous definitions and measurement approaches, the literature consistently shows higher rates of non-home discharge and greater reliance on post-acute care services among frail patients. Given this elevated risk, early coordination of structured post-acute support (e.g., inpatient rehabilitation or skilled nursing) may facilitate safer transitions of care; discharge destination has also been associated with readmission risk in arthroplasty populations [36].

Claims-based studies further demonstrate that frailty is independently associated with greater post-acute care utilization, including skilled nursing facility use and higher one-year healthcare expenditures following arthroplasty [32]. Imaging-defined sarcopenia has also been linked to poorer early functional status at discharge and greater reliance on assistive devices after THA, suggesting a need for prolonged or intensified postoperative PT [38]. Although PT frequency and intensity are inconsistently reported, studies incorporating functional recovery metrics show delayed ambulation milestones and worse early mobility among sarcopenic patients, supporting higher rehabilitation demand [31]. Collectively, these findings suggest that early identification of frailty and sarcopenia may improve prediction of PT needs, discharge planning, and allocation of rehabilitation resources, with potential to reduce avoidable post-acute utilization and healthcare costs.

## 6. Clinical Implications for Rehabilitation Planning

### 6.1. Preoperative Screening Feasibility

The findings synthesized in this review support the feasibility and clinical value of incorporating frailty and sarcopenia screening into preoperative assessment for patients undergoing THA and TKA. Simple, low-burden tools (e.g., CFS, mFI, SARC-CalF, grip strength, gait speed) can identify patients at increased risk for delayed functional recovery, non-home discharge, and greater post-acute utilization [37,39,40,41,42,43]. Importantly, these screening measures are practical in routine clinical workflows and do not require advanced imaging or specialized equipment, addressing a key barrier to widespread implementation. Evidence from both arthroplasty-specific cohorts and broader surgical populations suggests that frailty and sarcopenia capture biological vulnerability not fully explained by age, comorbidity burden, or procedure type alone [42,43,44].

Preoperative identification of frailty or sarcopenia may therefore allow rehabilitation teams to anticipate recovery challenges before surgery, rather than reacting to delayed progress postoperatively. For example, sarcopenia risk has been shown to predict delayed achievement of independent walking after THA [37], while frailty consistently predicts prolonged hospitalization and non-home discharge across multiple large datasets [40,41,42,43]. Routine screening may therefore improve prognostication, align expectations, and trigger early care coordination for post-acute needs. Screening may be most effectively performed at the initial surgical consultation or during formal preoperative assessment, allowing sufficient time for risk stratification, discharge planning, and potential prehabilitation interventions.

### 6.2. Stratified Rehabilitation Pathways

The consistent association between frailty, sarcopenia, and adverse recovery trajectories suggests a role for stratified rehabilitation pathways following THA and TKA. Rather than applying uniform postoperative PT protocols, rehabilitation intensity, duration, and setting may be better tailored according to preoperative vulnerability profiles. Frail or sarcopenic patients are more likely to require inpatient rehabilitation or skilled nursing facility placement and experience delayed mobility milestones, indicating higher rehabilitation demand [37,40,41,45]. Conversely, non-frail patients may safely progress through accelerated pathways with earlier discharge and outpatient-focused rehabilitation.

Importantly, stratification should not be interpreted as a rationale to limit access to surgery or rehabilitation. Studies of patient-reported outcomes demonstrate that even severely frail patients derive meaningful benefit from arthroplasty, although the magnitude of improvement may be attenuated [39]. Instead, stratified pathways may help optimize resource allocation by identifying patients who would benefit from earlier PT engagement, closer monitoring, or extended rehabilitation timelines. Incorporating frailty and sarcopenia into discharge prediction models may also improve coordination between surgical teams, physical therapists, and post-acute care services, reducing unplanned discharge delays and mismatches between patient needs and rehabilitation setting [40,41,42,43]. These findings are consistent with emerging literature emphasizing the importance of biological vulnerability in shaping postoperative recovery trajectories and healthcare utilization, further supporting the role of frailty and sarcopenia as clinically meaningful predictors beyond traditional risk factors [45]. To support clinical translation, rehabilitation considerations and discharge planning implications by frailty and sarcopenia profile are summarized in Table 3.

### 6.3. Implications for Prehabilitation and Early Rehabilitation

The identification of frailty and sarcopenia prior to surgery has important implications for prehabilitation and early postoperative rehabilitation strategies. Several studies highlight that sarcopenic patients enter surgery with reduced muscle strength, slower gait speed, and diminished functional reserve, all of which may limit their ability to fully engage in postoperative PT [30,46,47]. These findings suggest that targeted preoperative interventions, such as strength training, balance exercises, and nutritional optimization, may enhance physiological reserve and improve postoperative rehabilitation responsiveness, particularly in high-risk patients.

In the early postoperative period, awareness of frailty and sarcopenia status may guide PT goal setting and progression. Frail patients may require more gradual advancement of mobility tasks, additional assistive device training, and closer supervision to mitigate fall risk and prevent early setbacks [37,44]. Conversely, early identification of patients at risk for delayed recovery may justify proactive escalation of PT intensity or duration rather than reactive extension of care once progress stalls. While direct evidence linking frailty-guided rehabilitation strategies to improved outcomes remains limited, the existing literature strongly supports frailty and sarcopenia as clinically meaningful markers of rehabilitation capacity and recovery efficiency [44,45,48].

## 7. Knowledge Gaps and Future Directions

### 7.1. Inconsistent Definitions and Measurement Approaches

A major limitation across the existing literature is the substantial heterogeneity in how frailty and sarcopenia are defined and operationalized in arthroplasty populations. Studies included in this review employed a wide range of frailty instruments, including the CFS, mFI, electronic frailty index, and phenotypic measures, each capturing different dimensions of vulnerability and yielding variable prevalence estimates and predictive performance [39,40,41,42,43,44]. Similarly, sarcopenia has been assessed using diverse approaches ranging from simple screening tools (e.g., SARC-CalF, grip strength, gait speed) to imaging-based muscle mass indices derived from CT or DXA, often without concurrent strength or performance measures [30,37,46,47,48]. This lack of standardization limits cross-study comparability and complicates translation of findings into clinical rehabilitation pathways.

Future research would benefit from consensus-driven approaches to frailty and sarcopenia assessment in arthroplasty populations, with particular emphasis on measures that are feasible in routine clinical practice and most strongly associated with rehabilitation-relevant outcomes. Harmonization of definitions would enable more robust comparisons across studies and facilitate development of standardized risk stratification frameworks to guide rehabilitation planning.

### 7.2. Lack of Physical Therapy–Specific Utilization and Dose–Response Data

Despite consistent associations between frailty, sarcopenia, and adverse recovery trajectories, there remains a notable paucity of studies directly measuring physical therapy utilization. Most investigations infer rehabilitation demand through length of stay, discharge disposition, or early milestones rather than reporting PT-specific variables (e.g., frequency, duration, intensity, progression) [37,40,41,42,43,45]. It remains unclear whether differences reflect underdosing, reduced tolerance, or intrinsic limitations.

Addressing this gap will require prospective studies that integrate detailed rehabilitation metrics alongside frailty and sarcopenia assessments. Capturing PT dose–response relationships may clarify whether vulnerability-tailored rehabilitation strategies can mitigate disparities in recovery and inform procedure-specific recommendations for PT intensity and duration after THA and TKA.

### 7.3. Research Priorities and Future Directions

Key priorities include prospective evaluation of vulnerability-guided rehabilitation pathways, trials of prehabilitation (especially resistance training and nutritional optimization) in high-risk patients [30,46,47,48], and studies examining differences after THA versus TKA [37,45]. Implementation research is also needed to integrate vulnerability measures into multidisciplinary perioperative care models that include surgeons, physical therapists, and post-acute care teams. Understanding how vulnerability-informed rehabilitation planning can be operationalized in real-world settings will be critical to translating observational associations into meaningful improvements in patient-centered recovery after total joint arthroplasty.

## 8. Conclusions

Frailty and sarcopenia are important and complementary markers of biological vulnerability that influence functional recovery and physical therapy utilization after total hip and knee arthroplasty. This review highlights consistent associations between preoperative frailty and sarcopenia and delayed mobility, reduced independence inADL, non-home discharge, and greater post-acute care use—outcomes not fully explained by age, comorbidity burden, or procedure type alone. While frailty appears at least partially dynamic and may improve after joint replacement, sarcopenia reflects a more persistent, muscle-specific limitation that can constrain rehabilitation responsiveness without targeted intervention. Together, these constructs help explain heterogeneity in recovery trajectories and rehabilitation demand after arthroplasty.

The evidence supports incorporating feasible frailty and sarcopenia screening tools into preoperative assessment to improve discharge planning, tailor rehabilitation pathways, and optimize resource allocation. However, progress is limited by inconsistent definitions and a lack of PT-specific utilization and dose–response data. Future research should standardize vulnerability assessment and prospectively test vulnerability-guided rehabilitation strategies, including prehabilitation, to enable more personalized, efficient, and patient-centered rehabilitation after THA and TKA.

## Figures and Tables

**Table 1:** Frailty and Sarcopenia Assessment Tools Used in THA and TKA Populations.

Assessment Tool	Construct Assessed	Key Domains Captured	Typical Clinical Setting	Feasibility	Relevance to Rehabilitation Planning
Clinical Frailty Scale	Frailty	Global fitness, dependence, vulnerability	Preoperative clinic, inpatient	High (single-item, clinical judgment)	Predicts therapy tolerance, discharge destination, need for post-acute care
Modified Frailty Index	Frailty	Comorbidities, functional deficits	Preoperative assessment, databases	Moderate (chart-based)	Identifies patients at risk for prolonged LOS and delayed recovery
Fried Frailty Phenotype	Frailty	Weight loss, weakness, exhaustion, gait speed, activity	Research, select clinics	Moderate–low (multiple measures)	Highlights physical components affecting mobility and endurance
Electronic Frailty Index	Frailty	Accumulated health deficits	Administrative datasets	High (automated)	Useful for population-level risk stratification; limited bedside use
Grip Strength	Sarcopenia	Muscle strength	Preoperative clinic, rehab	High (handheld dynamometer)	Directly informs strength deficits targetable by PT
Gait Speed	Sarcopenia	Physical performance	Preoperative clinic, inpatient, rehab	High (minimal equipment)	Predicts mobility recovery and ambulation milestones
Chair Rise/Sit-to-Stand	Sarcopenia	Lower-extremity strength, power	Clinic, rehab	High	Relevant to transfers, stair negotiation, fall risk
SARC-F/SARC-F + Calf Circumference (SARC-CalF)	Sarcopenia (screening)	Strength, walking, rising, falls, muscle mass proxy	Preoperative clinic	Very high (questionnaire)	Rapid identification of patients at risk for poor rehab responsiveness
Imaging-based muscle mass	Sarcopenia	Muscle quantity, quality	Research, select centers	Low (cost, access)	Less directly actionable for PT planning; adjunctive risk marker

Abbreviations: THA, total hip arthroplasty; TKA, total knee arthroplasty; LOS, length of stay; SARC-CalF, Strength, Assistance with walking, Rise from a chair, Climb stairs, Falls with calf circumference; PT, physical therapy.

**Table 2:** Summary of Key Studies Examining Frailty or Sarcopenia and Postoperative Functional Recovery and PT Utilization After THA and TKA.

Author (Year)	Population	Vulnerability Measure	Outcomes Reported	Key Findings Relevant to Rehabilitation
Cooper et al. (2016) [16]	Orthopedic surgery (incl. THA/TKA)	Multiple frailty indices	LOS, discharge, mortality	Frailty predicted prolonged LOS and non-home discharge
Shin et al. (2016) [23]	THA/TKA	Simplified frailty index	Complications, LOS	Higher frailty associated with worse early outcomes
Schmucker et al. (2019) [3]	THA/TKA	Frailty (systematic review)	Short-term outcomes	Frailty consistently linked to poorer early recovery
Johnson et al. (2022) [28]	THA/TKA	Frailty transitions	ADL, frailty status	Frailty status improved postoperatively in many patients
Kappenschneider et al. (2024) [29]	THA/TKA	Frailty phenotype	Functional outcomes	Joint replacement improved frailty in OA patients
Ardeljan et al. (2022) [22]	TKA	Imaging-defined sarcopenia	LOS, complications	Sarcopenia associated with longer LOS and complications
Chang et al. (2023) [24]	THA	Sarcopenia	Complications, falls	Sarcopenia increased postoperative risk and impaired recovery
Tanaka et al. (2024) [31]	THA	Sarcopenia severity	Function, PROMs	Severe sarcopenia predicted worse 6-month function
Kim et al. (2025) [33]	THA rehab patients	Sarcopenia	Balance, gait	Sarcopenia associated with impaired gait independence
Wall et al. (2025) [35]	THA/TKA	CFS	Discharge destination	Higher frailty strongly predicted inpatient rehab use
Ron et al. (2025) [32]	THA/TKA	Frailty	Post-acute care, costs	Frailty linked to higher post-acute utilization and expenditures
Nanri et al. (2024) [37]	THA/TKA	Sarcopenia risk	Walking independence	Sarcopenia predicted delayed ambulation milestones

Abbreviations: THA, total hip arthroplasty; TKA, total knee arthroplasty; LOS, length of stay; ADL, activities of daily living; PROMs, patient-reported outcome measures; CFS, Clinical Frailty Scale.

**Table 3:** Rehabilitation Implications by Frailty and Sarcopenia Profile in THA and TKA.

Vulnerability Profile	Expected Recovery Challenges	Rehabilitation Considerations	Discharge Planning Implications
Non-frail, non-sarcopenic	Rapid early recovery; predictable mobility gains	Standard postoperative PT pathways; early mobilization and progression	High likelihood of home discharge with outpatient or home-based PT
Frail without sarcopenia	Reduced physiologic reserve; limited tolerance to therapy; higher risk of complications	Slower progression, attention to fatigue and safety; close monitoring of response to therapy	Increased likelihood of non-home discharge; early coordination with post-acute care services
Sarcopenic without frailty	Muscle weakness, impaired gait speed, delayed functional milestones	Emphasis on strength, balance, and task-specific training; close monitoring of mobility progression	Potential need for extended or intensified PT despite otherwise low medical risk
Combined frailty and sarcopenia	Multisystem vulnerability with pronounced functional impairment; highest risk for delayed recovery	Individualized, paced rehabilitation with focus on safety, endurance, and neuromuscular deficits	Highest likelihood of inpatient rehabilitation or skilled nursing facility placement
Pre-frail or mild sarcopenia	Subclinical vulnerability; risk of delayed recovery if unrecognized	Early identification and proactive rehabilitation engagement; potential role for prehabilitation	May benefit from early discharge planning despite apparent low risk

Abbreviations: THA, total hip arthroplasty; TKA, total knee arthroplasty; PT, physical therapy.

## Data Availability

No new datasets were generated or analyzed in this study; therefore, data availability is not applicable.
